# Retrospective analysis of nosocomial infections in the intensive care unit of a tertiary hospital in China during 2003 and 2007

**DOI:** 10.1186/1471-2334-9-115

**Published:** 2009-07-25

**Authors:** Ji-Guang Ding, Qing-Feng Sun, Ke-Cheng Li, Ming-Hua Zheng, Xiao-Hui Miao, Wu Ni, Liang Hong, Jin-Xian Yang, Zhan-Wei Ruan, Rui-Wei Zhou, Hai-Jiao Zhou, Wen-Fei He

**Affiliations:** 1 Department of Infectious Diseases, The Third Affiliated Hospital to Wenzhou Medical College, Rui'an, Zhejiang, 325200, PR China; 2 Department of Laboratories The Third Affiliated Hospital to Wenzhou Medical College, Rui'an, Zhejiang, 325200, PR China; 3 Department of Infection and Liver Diseases, The First Affiliated Hospital to Wenzhou Medical College, Wenzhou, Zhejiang, 325000, PR China; 4 Department of Infectious Diseases, Shanghai Changzheng Hospital, Shanghai, 200003, PR China; 5 Intensive Care Unit, The Third Affiliated Hospital to Wenzhou Medical College, Rui'an, Zhejiang, 325200, PR China; 6 Department of Hospital Infection Management, The Third Affiliated Hospital to Wenzhou Medical College, Rui'an, Zhejiang, 325200, PR China; 7 Department of Natural Products Chemistry, School of Pharmacy, Wenzhou Medical College, Wenzhou, Zhejiang, 325035, PR China

## Abstract

**Background:**

Nosocomial infections are a major threat to patients in the intensive care unit (ICU). Limited data exist on the epidemiology of ICU-acquired infections in China. This retrospective study was carried out to determine the current status of nosocomial infection in China.

**Methods:**

A retrospective review of nococomial infections in the ICU of a tertiary hospital in East China between 2003 and 2007 was performed. Nosocomial infections were defined according to the definitions of Centers for Disease Control and Prevention. The overall patient nosocomial infection rate, the incidence density rate of nosocomial infections, the excess length of stay, and distribution of nosocomial infection sites were determined. Then, pathogen and antimicrobial susceptibility profiles were further investigated.

**Results:**

Among 1980 patients admitted over the period of time, the overall patient nosocomial infection rate was 26.8% or 51.0 per 1000 patient days., Lower respiratory tract infections (LRTI) accounted for most of the infections (68.4%), followed by urinary tract infections (UTI, 15.9%), bloodstream (BSI, 5.9%), and gastrointestinal tract (GI, 2.5%) infections. There was no significant change in LRTI, UTI and BSI infection rates during the 5 years. However, GI rate was significantly decreased from 5.5% in 2003 to 0.4% in 2007. In addition, *A. baumannii, C. albicans *and *S. epidermidis *were the most frequent pathogens isolated in patients with LRTIs, UTIs and BSIs, respectively. The rates of isolates resistant to commonly used antibiotics ranged from 24.0% to 93.1%.

**Conclusion:**

There was a high and relatively stable rate of nosocomial infections in the ICU of a tertiary hospital in China through year 2003–2007, with some differences in the distribution of the infection sites, and pathogen and antibiotic susceptibility profiles from those reported from the Western countries. Guidelines for surveillance and prevention of nosocomial infections must be implemented in order to reduce the rate.

## Background

Nosocomial infections, also called healthcare acquired infections or health care-associated infections, is defined by the CDC as a localized or systemic condition resulting from an adverse reaction to the presence of an infectious agent(s) or its toxin(s), without any evidence that the infection was present or incubating at the time of admission to the acute care setting [1]. Nosocomial infections have become an important public health issue worldwide. Nosocomial infections may result in an excess length of stay in hospital for up to 10 days and an increase in the costs of hospitalization.[2,3] Nosocomial infections pose a critical threat to patients, especially in the high-risk departments, such as the Intensive Care Unit (ICU).[4,5] In industrialized countries, nosocomial infections occurs in 2–12% of hospitalized patients, with the rates being up to 21% in ICU, while the rates are 3–18% in hospitalized patients with the rates being up to 54% in ICU. [5-11] In China, there are more than 10,000 hospitals with significant differences among each other in the size, facilities, administration, teaching, research and academic levels. A few studies reported nosocomial infections in China, but these studies were limited in small sample sizes and short period of time, publication in Chinese journals, with or without English abstracts. [11-13] Recently, we carried out a retrospective study to determine the current status of nosocomial infections in a tertiary hospital in East China. All data on nosocomial infections between year 2003 and 2007 were retrieved and reviewed. The overall patient nosocomial infection rate, the incidence density rate of nosocomial infections, the excess length of stay, and distribution of nosocomial infection sites were determined. Then, pathogen and antimicrobial susceptibility profiles were further investigated.

## Methods

The study was performed in the Affiliated Hospital of Wenzhou Medical University, which situates in Zhejiang province, East China, with over 1000 beds and one mixed ICU of 13 beds. Since 1996, the hospital started an infection control program, including collection of data on infections acquired in the hospital. In the present study, data from January 2003 to December 2007 for patients in the ICU were retrieved by the Infection Control Team since completed raw data of the patients in ICU were available only after 2003. This retrospective study was approved by the Medical Ethics Committee of The Third Affiliated Hospital, Wenzhou Medical College.

All patients admitted to the ICU for more than 48 hours were monitored for nosocomial infections, which were defined according to the American CDC.[1] Infections developed within 48 hours of discharge from the ICU were also considered to be ICU-acquired unless there was an identified cause after discharge.

The major nosocomial infections, including lower respiratory tract infections (LRTIs), urine tract infections (UTIs), bloodstream infections (BSIs) and gastrointestinal tract infections (GIs) were defined as followings. LRTIs refer to lower respiratory tract infection, other than pneumonia, i.e. bronchitis, tracheobronchitis, bronchiolitis, tracheitis, without evidence of pneumonia. BSIs refers to laboratory-confirmed bloodstream infections, UTIs refers to symptomatic urinary tract infections and GIs refers to gastrointestinal tract (esophagus, stomach, small and large bowel, and rectum) infections excluding gastroenteritis and appendicitis. The detailed criteria to diagnose these nosocomial infections were described in the CDC documents [1].

Data on the date and site of infection, patient demographic information and device use were collected for each infection. Moreover, data on the isolated pathogens and their susceptibility testing to antimicrobial agents, if available, were also collected.

The overall patient nosocomial infection rate was calculated by dividing the total number of patients with nosocomial infections by the total number of patients in the ICU (×100) during the defined period of time (i.e. each year). The incidence density rate of nosocomial infections was calculated by dividing the total number of nosocomial infections by the total patient days (×1000) during the defined period of time. The total patients days were calculated by summating the days of each patient in the ICU. In the meantime, the length of stay, which was defined as the overall days of a patient spent in the hospital including the ICU and another department to which the patient was transferred from ICU after stabilization of the conditions. The excess length of stay was then calculated by subtracting the average length of stay for patients without nosocomial infection from that of patients with nosocomial infections.

Statistical analyses were performed using SPSS software version 13.2 (SPSS Inc., Chicago, Ill., USA). Chi-square test and Spearman's rank-correlation coefficients were applied where appropriate. For all analyses, a *P *value of less than 0.05 was considered statistically significant.

## Results

From 2003 to December 2007, medical data of 1980 patients discharged from the hospital ICU were colleted. The average length of stay was 9.95 days, giving 19700 patient-days. Among these patients, 531 patients acquired a total of 1005 nosocomial infections, including 125 patients with two infections, 33 patients with three infections, 12 patients with four infections and one patient with five infections (Table 1).

**Table 1:** Distribution of nosocomial infections by site

Infection site	Number (%) of nosocomial infections during the 5 years				
	
	2003	2004	2005	2006	2007
LRTIs	102 (70.3)	121 (71.6)	160 (67.8)	159 (69.4)	145 (64.2)
UTIs	24 (16.6)	28 (16.6)	40 (16.9)	25 (11.2)	43 (19.0)
BSIs	6 (4.1)	4 (2.4)	18 (7.6)	19 (8.3)	12 (5.3)
GIs	8 (5.5)	7 (4.1)	6 (2.6)	3 (1.3)	1 (0.4)
Surgical sites	1 (0.7)	1 (0.6)	2 (0.8)	4 (1.7)	2 (0.9)
Skin & soft tissue	2 (1.4)	2 (1.2)	1 (0.4)	1 (0.4)	3 (1.3)
Other sites	2 (1.4)	6 (3.5)	9 (3.9)	18 (7.7)	20 (8.9)
Total	145 (100)	169 (100)	236 (100)	229 (100)	226 (100)

BSIs, bloodstream infections; LRTIs, lower respiratory infections; UTIs, urinary tract infections; GIs, gastrointestinal infections.

The overall patient nosocomial infection rate was 26.8%, ranging from 22.6% to 33.2% among the 5 years. There was a significant difference in the infection rates among the 5 years (χ^2 ^= 10.395, *P *= 0.035). The incidence density rate of nosocomial infections was 51.0 per 1000 patient days, ranging from 38.5 to 59.2 among the 5 years. The excess length of stay was 9.4 days, ranging from 3.3 days in 2003 to 11.5 days in 2004 [see Additional file 1].

Lower respiratory tract infections (LRTIs) including bronchitis, tracheobronchitis and pneumonia, were the most common infections, occurring in 34.7% of the 1980 patients, followed by urine tract infections (UTIs) (8.1%), and bloodstream infections (BSIs) (3.0%). Among the 1005 nosocomial infections, LRTIs accounted for 68.4%, followed by UTIs (15.9%), BSIs (5.9%) and gastrointestinal tract infections (2.5%). Most (76.0%) patients with nosocomial LRTIs had received mechanical ventilation or tracheotomy before the infections, whereas 50.0% of nosocomial UTIs and 54.2% of nosocomial BSI were catheter associated (Table 1).

There is no significant change in LRTI, UTI and BSI rates during the 5 years. The GI infection rate was significantly decreased from 5.5% in 2003 to 0.4% in 2007 (χ^2 ^= 12.603, *P *= 0.012), whereas nosocomial infections in other sites was increased significantly (χ^2 ^= 12.858, *P *= 0.012). The nosocomial infection rates at the surgical sites and skin and soft tissues remained under 2% (Table 1).

Pathogens were isolated and identified from 530 (52.7%) of 1005 nosocomial infections, or, in 338 (63.7%) of the 531 patients. The isolated pathogens responsible for nosocomial infections differed among the infection sites (Table 2). In patients with LRTIs, *Acinetobacter baumannii *and *Klebsiella pneumoniae *were the most frequently isolated pathogens, followed by *Pseudomonas aeruginosa *and *Staphylococcus aureus*, accounting for more than half of the LRTI related pathogen population. In patients with UTIs, the fungi, especially *Candida albicans*, were the most common pathogens, followed by *Escherichia coli*. *Staphylococcus epidermidis*, *E. coli*, and *S. aureus *were the first three most common pathogens for BSIs. In addition, *A. baumannii *was commonly isolated in UTIs and BSIs (Table 2).

**Table 2:** Pathogens identified from patients with three major nosocomial infections during 2003–2007

Pathogen	LRTI (%) (n = 353)	UTI (%) (n = 84)	BSI (%) (n = 42)
*A. baumannii*	18.9	3.6	9.5
*K. pneurnoniae*	15.0	3.6	4.8
*P. aeruginosa*	11.3	1.2	9.5
*S. aureus*	11.0	0	14.3
*C. albicans*	9.1	33.3	2.3
*S. epidermidis*	5.9	3.6	19.0
*E. coli*	4.0	16.7	16.7
*B. cepacia*	4.0	0	2.3
Other candida	1.1	21.4	4.8

BSIs, bloodstream infections; LRTIs, lower respiratory infections; UTIs, urinary tract infections; GIs, gastrointestinal infections.

n, number of infections in which pathogens were isolated; %, the percentage of the pathogens in each of the three infections.

Data on susceptibility testing were available for 3328 isolates, including 195 isolates of *E. coli*, 359 isolates of *S. aureus*, and 549 isolates of *P. aeruginosa*. Overall, 79.3%, 80.0%, 82.3% and 77.8% of *E. coli *isolates were resistant to trimethoprim/sulfamethoxazole (TMP/SMX) and ciprofloxacin, cefotaxime, and amoxicillin/clavulanic acid, respectively. All *S. aureus *isolates were sensitive to vancomycin, but 24.0%, 37.9%, 68.1% and 89.6% of isolates were resistant to nitrofurantoin, TMP/SMX, rifampin and ciprofloxacin, respectively. In addition, 93.1%, 41.3% and 66.9% of *P. aeruginosa *were resistant to TMP/SMX, ciprofloxacin and levofloxacin, respectively [see Additional file 2].

All patients with nosocomial infections were treated with empirical antimicrobial therapies or according to the antimicrobial susceptibility test results, when available. The mortality rates in the patients with nosocomial infections were 20.2% (17/84), 12.2% (12/98), 15.1% (18/119), 15.1% (18/119) and 17.1% (19/111), respectively, in 2003, 2004, 2005, 2006 and 2007. However, based on the medical records, none of the mortalities were directly related to nosocomial infections.

## Discussion

In the present study, the overall patient nosocomial infection rate in the ICU was 26.8% during 2003 and 2007, which was higher than in the ICUs in many industrialized countries where the rates ranging from 7.7% to 16.5%, [14-16] and even higher than the rate (14.7%) observed in 55 ICUs of developing countries.[17] However, the rate is comparable with those reported in some Latin American countries such as Argentina and Brazil [10], and slightly lower than that reported in India. [18]

Over the 5 years, the lowest rate was reported in 2003 (22.6%), and the highest was reported in 2004 (33.2%). One plausible explanation is that in the early of 2003 the country was suffering from the outbreak of highly infectious pneumonia, namely severe acute respiratory syndrome (SARS). Due to the massive campaign to prevent the spread of SARS, nosocomial infections were indirectly reduced. Being fatigued from the campaign over the previous year, disinfection and sterilization procedures might be loosen in 2004, explaining the moderate rebound in 2004. However, the incidence density rate of nosocomial infections was the lowest in 2004, due to considerably longer stay of some patients in the ICU (Table 1).

The average length of stay in the hospital varied from 3.3 to 11.5 between 2003 and 2007, with the overall average being 9.4 days, which is generally in agreement with those (4.3–15.6 days) reported in European and the United States [19-21], but much less than that reported in Taiwan [22]. However, the it must be also mentioned that SARS outbreak had some impact on the overall length of stay. Although the average stay in the ICU was only 6.6 days, the shortest among the 5 years, the average stay in the hospital was the longest for both patients with and those without nosocomial infections due to the isolation policy imposed in the special period of time.

The distribution of nosocomial infections in the present study differed from that reported in the United States. We found that the LRTIs were the most common infections in the ICU, accounting for 68.4% of overall infections, whereas UTIs was the most frequently reported infections in the ICUs in the United States, with the rate of 31%, followed by pneumonia of 27%. [14] The proportions of UTIs and BSIs in the present study were relatively lower than the data reported in the United States (15.9% vs. 31% and 5.9% vs. 19%, respectively).[14] Although data from Europe revealed same three most common infection sites as the present study did, the absolute proportion of LRTIs was 47%, [6] which was lower than the rate in the present study. The common reasons proposed by studies in many Western countries have suggested that nosocomial LRTIs are mainly due to mechanical ventilation.[5,14] In China, air pollution, high density of population, and improper health habits such as smoking may also account for the high rate of LRTIs.

The rate of LRTIs slightly, but insignificantly, decreased from 2003 to 2007. There was no change in UTIs and BSIs rates during the 5 years. Notably, the GI rate significantly and stably decreased every year, suggesting an improvement in the environment and food sanitary in the region. Since the lower respiratory tract and the urinary tract were the first two sites that nosocomial infections frequently occurred in the ICU, constituting more than 80% of all nosocomial infections in 2003 and 2004, more efforts were later made to control these two kinds of infections, leading to decreased rates of lower respiratory tract and the urinary tract infections, and correspondingly increased rates of nosocomial infections at other sites.

The present study showed three quarters of LRTI patients received mechanical ventilation or tracheotomy, more than half of nosocomial UTI and BSI cases were catheter associated. These findings are consistent with previous studies, [4,9,10,14,23] and indicate that the nosocomial infections are often associated with the use of invasive device. Therefore, to effectively reduce nosocomial infections, the use of invasive device should be minimized and specific disinfection precautions taken during the device application.

In the present study, pathogens were isolated from 52.7% of overall nosocomial infections or 63.7% of all patients with nosocomial infections. Similar to the US report, Gram-negative bacteria accounted for 53.2% of the LRTIs in the present study, but the most frequently pathogens were *A. baumannii *and *K. pneurnoniae *in the present study whereas *P. aeruginosa *and *S. aureus *were the most common pathogens in the US report.[14] Consistent with the US report, fungi was the most frequently pathogen for nosocomial UTIs; *Candida *accounted for 54.7% of UTIs in the present study, suggesting a relatively narrow profile of pathogens in nosocomial UTIs. *S. epidermidis *was the most common pathogen for BSIs in both the present study and the US report; however, *E. coli*, instead of enterococci, was the second common pathogen in the present study. [14]

*E. coli *was the most common bacterial cause of nosocomial UTIs, and also frequently found in BSIs and LRTIs. It has been shown that the activity of beta-lactam antibiotics against *E. coli *is greatly reduced as a result of beta-lactamase production, but is restored by the addition of clavulanic acid.[24] In the present study, only 22.2% of *E. coli *isolates were sensitive to the formula amoxicillin combined with clavulanic acid, while 78.8% of *E. coli *isolates exhibited susceptibility to the combination in an UK study.[25] A high rate of resistance to TMP/SMX (79.3%) was observed in these *E. coli *isolates, in contrast to the rate of 40% reported in UK [26]. In addition, there was a high proportion (80.0%) of *E. coli *isolates resistant to ciprofloxacin, whereas the rate was less than 10% in UK and the United States.[26,27] These findings indicate that treatment with these antimicrobial agents for nosocomial infections caused by *E. coli *in China is likely to result in clinical failure in a substantial proportion of patients. We also found that a considerable number of *P. aeruginosa *isolates were resistant to fluoroquinolones, from 41.3% to ciprofloxacin to 66.9% to levofloxacin, which is comparable with the fluoroquinolone-resistant rates (49% to 64%) reported for patients in 55 ICUs of eight developing countries,[9] but much higher than that (30%) reported in the United States.[28] All *S. aureus *isolates in the present study were sensitive to vancomycin, which was similar to the observation by Tsuji et al in Japan.[29] In addition, we observed relatively low resistant rates to nitrofurantoin (24.0%) and TMP/SMX (37.9%), which renders these antimicrobial agents suitable for empirical treatment for *S. aureus *infections.

In China, The Guidelines for Surveillance and Prevention of Nosocomial Infections was established in 1994, and modified in 2000.[30,31] However, surveillance systems and control measures for nosocomial infections described in the guidelines were not completely implemented and executed in all hospitals, due to the imbalanced development and health care resources within the countries, and less attention to nosocomial infections in some hospitals. Therefore, it is believed that the nosocomial infection rates must be higher in some rural hospitals or even non-tertiary hospitals. In addition, due to empirical use or abuse of antibiotics, the proportion of antibiotic resistant pathogens for nosocomial infections in many lower level hospitals would also be higher than that reported in the present study.

It is noticed that although the mortality rates in patients nosocomial infections were between 15%–20%, there was no mortality directly caused by nosocomial infections. The major reasons for this observation would be the fact that refractory nosocomial infections are relatively less encountered based on our susceptibility testing, which showed that most pathogens were sensitive to many most commonly used antibiotics, indicating that they can be effectively controlled. Moreover, Zhejiang is one of richest provinces in China where medical and healthcare systems are relatively well established and anti-infectious therapies are not a big problems. Finally, it should be emphasized that our hospital is an tertiary infectious hospital with experience, methodologies and facilities to combat against various infections including nosocomial infections.

The present study has some limitations, due to the retrospective nature. First, data on risk factors, except for the use of the medical device, that are potentially associated with nosocomial infections were not available. These factors may include the primary diseases for admission to the ICU, patient resting posture (e.g. semirecumbent or supine body position), continuous prophylactic use of anti-peptic ulcer drugs, utilization of the alcohol-based handrubs and oral care, which need to be taken into consideration in the prospective studies. Second, the data on the identification and isolation of the pathogens and their susceptibility were available only for half of the nosocomial infections. It would produce more accurate data if these numbers were increased. Finally, the data on the clinical consequences were not available for most cases, making it impossible to compare the clinical outcomes between patients with and those without nosocomial infections. However, the present study was able to show that the length of stay in the hospital was significantly increased in patients with nosocomial infections, compared with those without the infections.

## Conclusion

In conclusion, there was a high and relatively stable rate of nosocomial infections in the ICU of a tertiary hospital in China through year 2003–2007, with some differences in the distribution of the infection sites, and pathogen and antibiotic susceptibility profiles from those reported in the Western countries. The Guidelines for Surveillance and Prevention of Nosocomial Infections must be implemented national wide in order to reduce the rate.

## Competing interests

The authors declare that they have no competing interests.

## Authors' contributions

J-GD, Q-FS and K-CL were the principal investigators who designed and conducted the study, analyzed the data, performed literature research and prepared the manuscript. M-HZ, X-HM and WN participated in the design of the study, contributed to the data analysis and made constructive comments on the manuscript. LH, J-XY, Z-WR, R-WZ, H-JZ and W-FH participated in the design of the study, collected the all required original data, and generated results and tables that were the basis of the manuscript. All authors have read and approved the final manuscript.

## Pre-publication history

The pre-publication history for this paper can be accessed here:

http://www.biomedcentral.com/1471-2334/9/115/prepub

## Supplementary Material

Additional file 1 **Overall nosocomial infections (NI) in the intensive care unit (ICU) and the effect on the length of stay in the hospital between 2003 and 2007**. Table S1. Click here for file

Additional file 2 **Antibiotic resistance in organisms isolated during 2003 and 2007**. Table S2. Click here for file

## References

[B1] Garner JS Jarvis WR Emori TG Horan TC Hughes JM CDC definitions for nosocomial infections Am J Infect Control 1988 16 128 140 10.1016/0196-6553(88)90053-3 2841893

[B2] Craig CP Connelly S Effect of intensive care unit nosocomial pneumonia on duration of stay and mortality Am J Infect Control 1984 12 233 238 10.1016/0196-6553(84)90114-7 6566521

[B3] Kappstein I Schulgen G Beyer U Geiger K Schumacher M Daschner FD Prolongation of hospital stay and extra costs due to ventilator-associated pneumonia in an intensive care unit Eur J Clin Microbiol Infect Dis 1992 11 504 508 10.1007/BF01960804 1526233

[B4] Jarvis WR Edwards JR Culver DH Nosocomial infection rates in adult and pediatric intensive care units in the United States Am J Med 1991 91 185S 191S 10.1016/0002-9343(91)90367-7 1928163

[B5] Kampf G Wischnewski N Schulgen G Schumacher M Daschner F Prevalence and risk factors for nosocomial lower respiratory tract infections in German hospitals J Clin Epidemiol 1998 51 495 502 10.1016/S0895-4356(98)00012-2 9635998

[B6] Jarvis WR Selected aspects of the socioeconomic impact of nosocomial infections: morbidity, mortality, cost, and prevention Infect Control Hosp Epidemiol 1996 17 552 557 8875302 10.1086/647371

[B7] Vincent JL Bihari DJ Suter PM Bruining HA White J Nicolas-Chanoin MH Wolff M Spencer RC Hemmer M The prevalence of nosocomial infection in intensive care units in Europe. Results of the European Prevalence of Infection in Intensive Care (EPIC) Study. EPIC International Advisory Committee JAMA 1995 274 639 644 10.1001/jama.274.8.639 7637145

[B8] Fagon JY Novara A Stephan F Girou E Safar M Mortality attributable to nosocomial infections in the ICU Infect Control Hosp Epidemiol 1994 15 428 434 7963432 10.1086/646946

[B9] National Nosocomial Infections Surveillance (NNIS) System Report, data summary from January 1992 through June 2004, issued October 2004 Am J Infect Control 2004 32 470 485 10.1016/j.ajic.2004.10.001 15573054

[B10] Rosenthal VD Guzman S Orellano PW Nosocomial infections in medical-surgical intensive care units in Argentina: attributable mortality and length of stay Am J Infect Control 2003 31 291 295 10.1067/mic.2003.1 12888765

[B11] Liu K Li YX Feng Z Liu YH Prevalence rate of nosocomial infection in a general hospital Chin J Infect Control 2006 5 48 50

[B12] Qi XH Ou SZ Surveillance of risk factors for nosocomial infection in patients in intensive care units Chin J Infect Control 2005 4 142 144

[B13] Ren N Wen XM WU AH Study on the changing trends in national nosocomial infection transaction investigation results Chin J Infect Control 2007 6 16 18

[B14] Richards MJ Edwards JR Culver DH Gaynes RP Nosocomial infections in medical intensive care units in the United States Crit Care Med 1999 27 887 892 10.1097/00003246-199905000-00020 10362409

[B15] Legras A Malvy D Quinioux AI Villers D Bouachour G Robert R Thomas R Nosocomial infections: prospective survey of incidence in five French intensive care units Intensive Care Med 1998 24 1040 1046 10.1007/s001340050713 9840237

[B16] Dettenkofer M Ebner W Hans FJ Forster D Babikir R Zentner J Pelz K Daschner FD Nosocomial infections in a neurosurgery intensive care unit Acta Neurochir (Wien) 1999 141 1303 1308 10.1007/s007010050434 10672301

[B17] Rosenthal VD Maki DG Salomao R Moreno CA Mehta Y Higuera F Cuellar LE Arikan OA Abouqal R Leblebicioglu H Device-associated nosocomial infections in 55 intensive care units of 8 developing countries Annals Internal Med 2006 145 582 591 10.7326/0003-4819-145-8-200610170-00007 17043340

[B18] Agarwal R Gupta D Ray P Aggarwal AN Jindal SK Epidemiology, risk factors and outcome of nosocomial infections in a Respiratory Intensive Care Unit in North India J Infect 2006 53 98 105 10.1016/j.jinf.2005.10.021 16343637

[B19] Díaz Molina C García Martín M Bueno Cavanillas A López Luque A Delgado Rodríguez M Gálvez Vargas R The estimation of the cost of nosocomial infection in an intensive care unit Med Clin (Barc) 1993 100 329 332 8455410

[B20] Dominguez TE Chalom R Costarino AT Jr The impact of adverse patient occurrences on hospital costs in the pediatric intensive care unit Crit Care Med 2001 29 169 174 10.1097/00003246-200101000-00033 11176179

[B21] Miller ZC Excess length of stay, charges, and mortality attributable to medical injuries during hospitalization JAMA 2003 290 1868 1874 10.1001/jama.290.19.2542-b 14532315

[B22] Chen YY Chou YC Chou P Impact of nosocomial infection on cost of illness and length of stay in intensive care units Infect Control Hosp Epidemiol 2005 26 281 7 10.1086/502540 15796281

[B23] Edwards JR Peterson KD Andrus ML Tolson J Goulding J Dudeck M Mincey R Pollock D Horan T National Healthcare Safety Network (NHSN) Report, data summary for 2006, issued June 2007 Am J Infect Control 2006 35 290 301 10.1016/j.ajic.2007.04.001 17577475

[B24] Brogden RN Carmine A Heel RC Morley PA Speight TM Avery GS Amoxycillin/clavulanic acid: a review of its antibacterial activity, pharmacokinetics and therapeutic use Drugs 1981 22 337 362 10.2165/00003495-198122050-00001 7037354

[B25] Farrell DJ Morrissey I De Rubeis D Robbins M Felmingham D A UK multicentre study of the antimicrobial susceptibility of bacterial pathogens causing urinary tract infection J Infect 2003 46 94 100 10.1053/jinf.2002.1091 12634070

[B26] Bean DC Krahe D Wareham DW Antimicrobial resistance in community and nosocomial *Escherichia coli *urinary tract isolates, London 2005–2006 Ann Clin Microbiol Antimicrob 2008 7 13 18564430 10.1186/1476-0711-7-13 PMC2440378

[B27] Peterson J Kaul S Khashab M Fisher A Kahn JB Identification and pretherapy susceptibility of pathogens in patients with complicated urinary tract infection or acute pyelonephritis enrolled in a clinical study in the United States from November 2004 through April 2006 Clin Ther 2007 29 2215 2221 10.1016/j.clinthera.2007.10.008 18042477

[B28] Slama TG Gram-negative antibiotic resistance: there is a price to pay Crit Care 2008 12 S4 18495061 10.1186/cc6820 PMC2391261

[B29] Tsuji BT Rybak MJ Cheung CM Amjad M Kaatz GW Community- and health care-associated methicillin-resistant Staphylococcus aureus: a comparison of molecular epidemiology and antimicrobial activities of various agents Diagn Microbiol Infect Dis 2007 58 41 47 10.1016/j.diagmicrobio.2006.10.021 17300912

[B30] Anonymous Guidelines for nosocomial infections control and prevention (Draft) Document 36, October 1994. Beijing, Ministry of Health, People's Republic of China

[B31] Anonymous Guidelines for nosocomial infections control and prevention (Draft) Document 431, November 2000. Beijing, Ministry of Health, People's Republic of China

