# Supplementary material for: Retrospective analysis of nosocomial infections in the intensive care unit of a tertiary hospital in China during 2003 and 2007
Source: BMC Infect Dis. 2009 Jul 25;9:115. doi: 10.1186/1471-2334-9-115 (PMC2722662; doi:10.1186/1471-2334-9-115)
Supplement: Additional file 1 — Overall nosocomial infections (NI) in the intensive care unit (ICU) and the effect on the length of stay in the hospital between 2003 and 2007. Table S1. [file 1471-2334-9-115-S1.doc]

**Additional file 1**. Overall nosocomial infections (NI) in the intensive care unit (ICU) and the effect on the length of stay in the hospital between 2003 and 2007

| Year | ICU discharge (n) | Patients with NI  (n) | Patients without NI  (n) | Patient NI rate (%) | Overall patient-days (average days) in ICU | Number of NIs | Incidence density rate (per 1000) | Average length of stay in hospital in cases with NI (days) | Average length of stay in hospital in cases without NI (days) | Excess length of stay in hospital (days) |
| --- | --- | --- | --- | --- | --- | --- | --- | --- | --- | --- |
| 2003 | 372 | 84 | 288 | 22.6 | 2447 (6.6) | 145 | 59.2 | 42.1 | 38.8 | 3.3 |
| 2004 | 295 | 98 | 197 | 33.2 | 4392 (14.9) | 169 | 38.5 | 39.4 | 27.9 | 11.5 |
| 2005 | 459 | 119 | 340 | 25.9 | 4202 (9.2) | 236 | 56.2 | 34.5 | 23.1 | 11.4 |
| 2006 | 461 | 119 | 342 | 25.8 | 4299 (9.3) | 229 | 53.3 | 35.3 | 24.5 | 10.8 |
| 2007 | 393 | 111 | 282 | 28.2 | 4360 (11.1) | 226 | 51.8 | 36.1 | 25.8 | 10.3 |
| Total | 1980 | 531 | 1449 | 26.8 | 19700 (9.95) | 1005 | 51.0 | 37.1 | 26.7 | 9.4 |
