# Supplementary material for: Retrospective analysis of nosocomial infections in the intensive care unit of a tertiary hospital in China during 2003 and 2007
Source: BMC Infect Dis. 2009 Jul 25;9:115. doi: 10.1186/1471-2334-9-115 (PMC2722662; doi:10.1186/1471-2334-9-115)
Supplement: Additional file 2 — Antibiotic resistance in organisms isolated during 2003 and 2007. Table S2. [file 1471-2334-9-115-S2.doc]

**Additional file 2**. Antibiotic resistance in organisms isolated during 2003 and 2007

| Antibiotic | Resistance rate (%) in the organism | | | | | | | |
| --- | --- | --- | --- | --- | --- | --- | --- | --- |
|  | *E. coli* | *K. pneurnoniae* | *A. baumannii* | *P. aeruginosa* | *B. cepacia* | *S. aureus* | *S. epidermidis* | *C. albicans* |
| Amoxicillin/clavulanic | 77.8 | 34.1 | - | - | - | - | - | - |
| Piperacilline-tazobactam | 21.2 | 18.6 | 49.2 | 8.3 | - | - | - | - |
| Cefotaxime | 82.3 | 26.4 | 85.9 | - | - | - | - | - |
| Ceftazidime | 61.9 | 20.2 | 43.8 | 21.5 | 16.9 | - | - | - |
| Trimethoprim/sulfamethoxazole | 79.3 | 39.7 | 51.7 | 93.1 | 22.5 | 37.9 | 69.9 | - |
| Amikacin | 23.9 | 10.6 | 46.4 | 8.3 | - | 60.8 | 53.2 |  |
| Levofloxacin | 75.0 | 31.1 | 56.5 | 66.9 | 10.3 | 81.2 | 84.3 | - |
| Ciproﬂoxacin | 80.0 | 23.4 | 56.3 | 41.3 | - | 89.6 | 85.0 | - |
| Imipenem | 0.0 | 0.0 | 9.1 | 23.0 | - | - | - | - |
| Nitrofurantoin | 14.7 | 37.5 | - | 27.7 | - | 24.0 | 69.9 |  |
| Penicillin | - | - | - | - | - | 100.0 | 97.8 | - |
| Methicillin | - | - | - | - | - | 81.0 | 90.7 | - |
| Rifampin | - | - | - | - | - | 68.1 | 63.1 | - |
| Vancomycin | - | - | - | - | - | 0.0 | 0.0 | - |
| Fluconazole | - | - | - | - | - | - | - | 6.1 |
| Itraconazole | - | - | - | - | - | - | - | 6.4 |
| 5-Fluorocytosine | - | - | - | - | - | - | - | 1.2 |
| Amphotericin B | - | - | - | - | - | - | - | 0.0 |
| Note: -, Not tested. | | | | | | | | |
